# Integration of Molecular Information in Risk Assessment of Patients with Myeloproliferative Neoplasms

**DOI:** 10.3390/cells10081962

**Published:** 2021-08-02

**Authors:** Giuseppe G. Loscocco, Giacomo Coltro, Paola Guglielmelli, Alessandro M. Vannucchi

**Affiliations:** Centro di Ricerca e Innovazione Malattie Mieloproliferative (CRIMM), Department of Experimental and Clinical Medicine, Azienda Ospedaliera Universitaria Careggi, University of Florence, Largo Brambilla 3, 50134 Florence, Italy; gloscocco@unifi.it (G.G.L.); giacomo.coltro@unifi.it (G.C.); paola.guglielmelli@unifi.it (P.G.)

**Keywords:** myeloproliferative neoplasms, polycythemia vera, essential thrombocythemia, myelofibrosis, *JAK2*, *CALR*, *MPL*, JAK-STAT pathway, additional mutations, prognosis

## Abstract

Philadelphia chromosome-negative myeloproliferative neoplasms (MPN) are clonal disorders of a hematopoietic stem cell, characterized by an abnormal proliferation of largely mature cells driven by mutations in *JAK2*, *CALR*, and *MPL*. All these mutations lead to a constitutive activation of the JAK-STAT signaling, which represents a target for therapy. Beyond driver ones, most patients, especially with myelofibrosis, harbor mutations in an array of “myeloid neoplasm-associated” genes that encode for proteins involved in chromatin modification and DNA methylation, RNA splicing, transcription regulation, and oncogenes. These additional mutations often arise in the context of clonal hematopoiesis of indeterminate potential (CHIP). The extensive characterization of the pathologic genome associated with MPN highlighted selected driver and non-driver mutations for their clinical informativeness. First, driver mutations are enlisted in the WHO classification as major diagnostic criteria and may be used for monitoring of residual disease after transplantation and response to treatment. Second, mutation profile can be used, eventually in combination with cytogenetic, histopathologic, hematologic, and clinical variables, to risk stratify patients regarding thrombosis, overall survival, and rate of transformation to secondary leukemia. This review outlines the molecular landscape of MPN and critically interprets current information for their potential impact on patient management.

## 1. Introduction

In 1951, William Dameshek coined the term “myeloproliferative disorders” to include what are currently known as classic Philadelphia chromosome-negative myeloproliferative neoplasms (MPN) [1]. The latest 2016 World Health Organization (WHO) classification of myeloid neoplasms includes four diseases: polycythemia vera (PV); essential thrombocythemia (ET); and myelofibrosis, either overt or prefibrotic primary myelofibrosis (PMF) [2]. The original breakthrough, Dameshek’s argument was that they might represent a relatively homogeneous family of clinically heterogeneous disorders that were expression of, and were caused by, an abnormal proliferation capability of a stem/progenitor cell in the bone marrow. Indeed, each has unique clinical and hematologic presentation, yet they are largely phenotypically overlapping, especially at presentation, suggesting that they might represent a continuum of a single disease. Not formally recognized in the WHO diagnostic criteria are those forms of MF that develop as progression of prior PV and ET (post PV-MF and post ET-MF, also collectively known as “secondary” MF). All MPN can transform into secondary acute myeloid leukemia, referred to as MPN-blast phase (BP), which is typically refractory to conventional chemotherapy and has dismal prognosis.

MPN are considered rare hematologic neoplasms, with an incidence that varies from 0.1 to 2.8/100,000 European inhabitants per year [3]; prevalence remains difficult to determine, but it may be anticipated to be progressively rising. In fact, the survival of patients with MPN is slowly but definitely improving in the last few decades, thanks to earlier diagnosis (nowadays facilitated by the inclusion of driver MPN-associated mutations among the WHO diagnostic criteria); better risk stratification (largely thanks to the knowledge of the genomic structure, including but not limited to driver mutations); and introduction of novel therapies, including JAK inhibitors, as well as allogeneic hematopoietic stem cell transplant (allo-HSCT).

There has been a tremendous accumulation of information deriving by the sequencing of DNA of MPN patients, which started in 2005 with the description of a point mutation in *JAK2* (V617F), marking more than 60% of all MPN, soon followed by discovery of *JAK2* exon 12, *MPL*, and *CALR* mutations. Their high frequency among MPN (overall, > 85%) and relative selectivity compared to other hematologic neoplasia warranted a revision of the diagnostic criteria, ultimately leading to current 2016 WHO classification. Then, not without surprise, the appreciation of an unexpected degree of genomic abnormalities complexity in MPN arose following the extensive use of next-generation sequencing, and opened the way to the generation of novel, more powerful risk stratification systems, particularly in MF. The appreciation of the uniform involvement of the JAK-STAT signaling pathway in MPN promoted the development of a new class of drugs, the JAK inhibitors, which although lacking selectivity against the mutated protein have nonetheless dramatically changed the life of patients, particularly MF and PV, and may be also carrying some advantages in terms of survival (MF) and reduction of thrombotic events, the leading cause of morbidity and mortality in PV.

## 2. Mutational Landscape of Myeloproliferative Neoplasms

### 2.1. Driver Mutations in JAK2, MPL, and CALR

Constitutive activation of the JAK-STAT signaling is the hallmark of all MPN and is sustained by somatic mutations in driver genes including *JAK2*, *MPL*, and *CALR*. The JAK-STAT pathway is critically involved in the regulation of cytokine- and growth factor receptor-mediated effects, as well as cell growth, survival, and differentiation of hematopoietic and immune compartments. Dysregulation of the JAK-STAT pathway confers cytokine independence and/or hypersensitivity to mutated cells, resulting in a survival advantage [4]. Somatic mutations in *JAK2*, *MPL,* and *CALR* are referred as “driver mutations” on the basis of their role in driving the MPN clinical phenotype and are crucial for the diagnosis along with selected laboratory and histopathological features. These mutations, documented in up to 85% of MPN patients, are mutually exclusive, but anecdotal cases of co-occurrence have been described in some reports [5,6]. Conversely, patients with features of MPN without any drive mutations are referred to as “triple negative” (TN) [7].

Two different types of *JAK2* mutations are described in MPN. The first type, discovered in 2005 by four different groups, is a single nucleotide variant at codon 617 (in exon 14) resulting in a valine to phenylalanine substitution [8,9,10,11]; the second type, described in 2007, comprises different changes in exon 12, particularly in-frame insertions or deletions in the region between codons 536 and 544, collectively defined as exon 12 mutations [12,13]. *JAK2*V617F is detected in approximately 95% of polycythemia vera (PV) and 50–60% of essential thrombocythemia (ET) and primary myelofibrosis (PMF), whereas *JAK2* exon 12 mutations are found exclusively in 2–3% of PV patients lacking the more common *JAK2*V617F. Patients with exon 12 mutations are characterized by erythroid-dominant myeloproliferation with a consequent lower leukocyte and platelet count at diagnosis, subnormal serum erythropoietin levels, a subtle tri-lineage hyperplasia in the bone marrow, and younger age at diagnosis in comparison to those with V617F mutation. However, there are no substantial prognostic differences between *JAK2* exon 12 and *JAK2* V617F-mutated PV patients [14,15].

The *JAK2*V617F mutation is capable of driving all the different MPN through ligand-independent activation of erythropoietin (EPO), thrombopoietin (TPO), and granulocyte-colony stimulating factor (G-CSF) tyrosine-kinase receptors. This constitutive activation results in an abnormal trilinear myeloproliferation [16,17,18]. Functionally, the inhibitory effect of the JAK2 pseudokinase domain is abrogated and the autonomous activation of intracellular signaling promotes cell proliferation, differentiation, and survival through downstream signaling molecules, including signal transducers and activators of transcription (STATs) [19], mitogen-activated protein kinases (MAPKs), phosphoinositol-3-kinase (PI3K), and mammalian target of rapamycin (mTOR) [20]. Although the question how *JAK2*V617F may induce different MPN is still incompletely answered to, the different clinical phenotypes probably reflect different host characteristics, different progenitor cell stage at which the mutation arises, the presence and order of additional variants, and the influence of bone marrow microenvironment. Moreover, *JAK2*V617F allele burden has been associated with different phenotype of MPN; homozygous status or a high mutant allele burden is found in most PV patients, and is associated with increased risk of thrombosis and fibrotic evolution [21,22], whereas ET and PMF patients tend to have a lower burden [23].

In 2006, mutations in the TPO receptor MPL, which specifically regulates megakaryopoiesis and platelet production through the JAK-STAT pathway, were discovered [24]. Mutations in *MPL* are found in 3–8% of ET and PMF and consist in gain of function variants at tryptophan 515 (W515) in exon 10 [25]. The most common point mutations are W515L and W515K. Other rare mutations at the same position include W515R, W515A, and W515G [26]. Although the *MPL*S505N variant was identified previously as a germline mutation in cases of familial thrombocythemia [27], it can also be acquired as a somatic event. Recently, many second-site mutations that enhance S505N-driven activation were described [28].

Finally, in 2013, two independent groups reported a breakthrough discovery by identifying a novel driver mutation in a large part of *JAK2* and *MPL* wild-type MPN patients. By applying whole exome sequencing, both groups identified somatic mutations in *CALR* encoding for calreticulin, a 46-kDa endoplasmic reticulum (ER) chaperone protein. CALR has a key role in the quality control of glycoprotein folding and maintenance of cell calcium homeostasis given its negatively charged *C*-terminus [29]. To date, more than 50 different mutations in *CALR* exon 9 have been described in approximately 20–25% of ET and 25–30% of PMF cases but not in PV patients [30,31]. The two most frequent *CALR* mutations, accounting for approximately 80% of all the mutations, include a type-1 52-bp deletion (c.1092_1143del; p.L367fs*46) and a type-2 5-bp insertion (c.1154_1155insTTGTC; p.K385fs*47). Atypical mutations are grouped as type 1-like and type 2-like in relation to structural similarities. All *CALR* mutations lead to a +1 frameshift of the open reading frame, resulting in a mutant protein lacking the ER-retention motif (KDEL) and containing a novel *C*-terminal chain of positively charged amino acids instead of the negatively charged amino acids of wild-type CALR. *CALR* subtypes contribute to determine clinical phenotype and outcomes in MPN. In PMF, type-1/1-like mutations are more prevalent than type-2/2-like (70% vs. 13%), while in ET they are more equally distributed (51% vs. 39%) [32]. In ET patients, type-1/1-like mutations were associated with a fibrotic phenotype and a significantly higher risk of myelofibrotic transformation, whereas type-2/2-like mutations were associated with higher platelet count and a lower risk of thrombosis [33]. In a large series of ET patients, both *CALR* variants correlated with a higher platelet count, lower hemoglobin, and leukocyte count compared to mutant *JAK2* [34]. Moreover, type-1/1-like mutations have been associated with a favorable survival in PMF [35]. Intense research efforts lead to elucidation of the mechanism underlying the oncogenic activity of mutated *CALR* in MPN. Mutant CALR binds to MPL within the ER, resulting in the constitutively activation of the JAK-STAT signaling [36,37,38,39]. Moreover, the MPL–CALR complex has been detected on cell surface, and its translocation appeared essential for the oncogenic activity [40,41]. More recently, a novel intriguing mode of action of mutated CALR was described suggesting an interleukin-6-dependent and TPO-MPL-independent mechanism [42].

### 2.2. Triple-Negative MPN

Mutations in *JAK2* exon 14 and exon 12, *CALR* exon 9, and *MPL* exon 10 account for over 90% of all MPN cases. These mutations are mutually exclusive, although rare cases of coexisting driver mutations are reported [5]. However, approximately 15% of ET and less than 10% of PMF patients lack a known driver mutation and are accordingly referred to as TN [43]. These cases of MPN require a particularly diligent diagnostic workup since reactive causes mimicking MPN-like disorders as well as alternative myeloid malignancies need to be carefully excluded. Moreover, it was reported that some TN cases tested positive for canonical somatic driver mutations at low mutant burden when re-sequenced using methodologies with a higher sensitivity [44,45,46].

At least 10% of TN ET and PMF patients harbor mutations outside of the canonical *MPL* and *JAK2* hotspots. Non canonical *MPL* mutations include T119I, S204F/P and E230G in the extracellular domain and Y591D/N in the intracellular domain [45], whereas non canonical *JAK2* mutations include V625F, F556V, R683G, and E627A [46]. As reported in functional studies, most of these genomic variants lead to a constitutive activation of the JAK-STAT signaling [45,46]. Some of them are exclusively described as somatic events, while others also represent germline variants, with the possibility that these patients may have a non-clonal erythrocytosis or thrombocytosis. These findings impose important implications for the diagnosis and management of TN patients, including unique therapeutic approach and eventually the screening of family members. Triple negative ET is an indolent disease with low incidence of vascular events and progression to MF, and is usually diagnosed in young, female patients. On the contrary, TN MF is a more aggressive disease with an adverse prognosis and a high rate of progression to blastic phase (BP).

### 2.3. Somatic Additional Mutations in Genes Frequently Involved in Myeloid Neoplasms

In last years, high-throughput next-generation sequencing (NGS) approaches resulted in the identification of a considerable number of additional somatic mutations with a prognostic and therapeutic value. At least one additional mutation can be found in more than 50% of patients, and the prevalence increases with patient age. These co-occurring mutations are not restricted to MPN, but occur also in other myeloid malignancies including acute myeloid leukemia (AML), myelodysplastic syndromes (MDS), and MDS/MPN overlap diseases, as well as in healthy individuals in the context of age-related clonal hematopoiesis (ARCH/CHIP) [44,47]. Additional genomic abnormalities affect genes involved in DNA methylation (*TET2*, *DNMT3A*, *IDH1*, and *IDH2*), histone modification (*ASXL1*, *EZH2*), mRNA splicing (*SFRB1*, *SRSF2*, *U2AF1*, and *ZRSR2*), signaling pathways (*LNK*/*SH2B3*, *CBL*, *NRAS*, *KRAS*, and *PTPN11*), and transcription factors (*RUNX1*, *NFE2*, *TP53*, and *PPM1D*).

The *TET2* gene encodes the second member of the TET proteins family, which are involved in the stepwise oxidation of 5methylcytosine (5mC) to 5-hydroxyymethylcytosine (5hmC), an important process in stem cell gene regulation. Loss of function mutations in *TET2* are mostly heterozygous and occur in 10–15% of MPN patients and 20–25% of BP. They are the most common co-occurring mutations in *JAK2*V617F-mutated MPN, although without a clear prognostic effect [48,49,50]. Recently, it has been reported that the order of acquisition of *JAK2* and *TET2* mutations may influence MPN phenotype, with “*JAK2*-first” more commonly detected in PV and “*TET2*-first” in ET patients [51]. DNMT3A belongs to a family of highly conserved DNA methyltransferases involved in either de novo DNA methylation or maintenance of pre-existing methylation patterns throughout cell division. *DNMT3A* mutations are frameshift/nonsense, resulting in reduced methyltransferase activity, and occur in 5–15% of MPN patients, with lower frequency in PV and ET compared to PMF and BP [48,49,50]. Similarly to *TET2*, MPN patients are more likely to present with PV when *JAK2*V617F is acquired before *DNMT3A* mutation, while patients who first acquired *DNMT3A* mutations more commonly develop an ET phenotype [52]. The *IDH1* and *IDH2* genes encode two additional epigenetic regulators involved in both DNA methylation and histone modification. Mutations in isocitrate dehydrogenase genes (*IDH1* and *IDH2*) occur mostly as point missense mutations at residues R132 in *IDH1* and R140 or R172 in *IDH2*. The mutant protein acquires the ability to convert alpha-ketoglutarate (a-KG) to 2-hydroxyglutarate (2-HG), favoring leukemogenesis through epigenetic dysregulation of a number of other genes. *IDH1* and *IDH2* mutations have been associated with worse prognosis in PMF with a higher risk of transformation to BP. They are reported in up to 6% of MPN and 30% of BP [53,54].

The *ASXL1* and *EZH2* genes encode for two important chromatin-binding protein of the polycomb family involved in the epigenetic regulation of gene expression through the interaction with polycomb repressive complex 1 (PRC1), polycomb repressive complex 2 (PRC2), and other transcription activators and repressors [55]. Loss of ASXL1 function results from nonsense and frameshift mutations in exon 12 and are more common in PMF and BP (18–37%) compared to PV and ET (5–10%) [54,56]. *ASXL1* mutations are associated with a worse prognosis in PV and PMF patients [48,57,58], and this unfavorable impact is not overcome by allogenic hematopoietic stem cell transplantation (allo-HSCT) [59]. Of interest, it has been recently reported that *ASXL1* mutations are frequently acquired during ruxolitinib treatment as part of clonal evolution of the disease [60]. *EZH2* mutations are less frequent than those in *ASXL1*, being found in up to 10% and 15% of MPN and BP patients, respectively. EZH2 seems to act as a tumor suppressor in MPN, and a high number of loss of function mutations have been identified that synergizes with *JAK2*V617F in initiating MPN and promoting myelofibrosis [61]. In PMF, *EZH2* mutations correlate with a higher leukocyte count, blast count, and larger spleen size at diagnosis. Moreover, *EZH2* mutations are an independent poor prognostic factor for reduced overall survival [62].

Acquired mutations in genes encoding for spliceosome proteins and other regulatory splicing factors are reported in all myeloid malignancies, particularly MPN and MDS, and mainly affect *SRSF2*, *U2AF1*, *ZRSR2*, and *SF3B1* genes. Spliceosome-affecting mutations are typically mutually exclusive and mostly occur as heterozygous missense mutations that confer a dominant negative activity affecting RNA splicing [63]. The *SRSF2* gene is the most frequently mutated, with variants mainly occurring at the Proline 95 residue. While being infrequent in PV and ET patients, *SRSF2* mutations occur in 8–22% of PMF and BP, where predict for poor overall and leukemia-free survival [56,64,65,66,67]. *U2AF1* mutations, mainly at the hotspot residues Serine 34 and Glutamine 157, occur in 5–15% of PMF and BP and are associated with a significantly shorter overall survival [68]. SF3B1 mutations affect exons 14–16, particularly the Lysine 700 amino acid, and are more commonly found in PMF and BP (up to 10%). Although rare in PV and ET, *U2AF1* and *SFRB1* mutations were associated with an inferior myelofibrosis-free survival compared to the with wild-type counterpart [48]. Finally, *ZRSR2* mutations are very rare in MPN and are more frequently identified in PMF, without a clear impact on prognosis.

LNK/SH2B3 is an adaptor protein that inhibits signaling through cytokine and tyrosine kinase receptors, including JAK2 [69,70]. Mutations affecting *LNK*/*SH2B3* are mostly missense substitutions and can be found in up to 10% of MPN and BP patients [48,49,50]. Although somatic *LNK* mutations detected in MPN patients are sparce through the gene, the *LNK*E208Q variant is the only one that has been identified both as germline variant in idiopathic erythrocytosis and acquired mutation in MPN, either alone or in association with a driver mutation [71]. Germline *LNK*E208Q mutation has been reported also in cases of familial MPN [72,73].

Mutations in oncogenic RAS pathway genes (*NRAS*, *KRAS*, *CBL*, *NF1*, *PTPN11*) are overall rare in MPN patients. *CBL* encodes for a multifunctional adaptor protein with ubiquitin ligase activity, and mutations are associated with stabilization of receptor tyrosine kinases, resulting in constitutive activation of signaling pathways, cytokine hypersensitivity, and autonomous cell proliferation [74]. *CBL* mutations are mostly missense substitutions and can be detected in up to 6% of PMF and BP patients [49,50]. Missense mutations in *NRAS*/*KRAS*, particularly in codons 12, 13, and 61, lead to constitutive activation of growth signaling [75], are rare in PMF (up to 5%), and are reported in up to 15% of BP [48,49]. In a recent paper, *CBL*/*NRAS*/*KRAS* mutations in PMF patients were associated with adverse clinical features, shorter overall survival, and poor response to JAK inhibitor therapy [76]. *PTPN11* mutations are found in up to 8% of BP and are associated with a reduced overall survival [50].

Among transcription factors, *RUNX1* and *NFE2* are the most commonly mutated genes in MPN. *RUNX1* mutations, result in protein inactivation, occur in up to 10% of BP, and are associated with a shortened survival [44]. The *NFE2* gene encodes for an important regulator of megakariocytopoiesis and/or erythropoiesis. Mutations are heterogenous but mainly represented by frameshift variants and deletions [77]. Although overall rare, *NFE2* mutations are enriched in PV (8% of cases). In one study the authors failed to find meaningful hematological and clinical correlates, nor a clear prognostic value [78], while in another study including a larger cohort of MPN patients, *NFE2* mutations adversely affected prognosis, increasing the risk of BP progression independently of age and other co-occurring mutations [79]. TP53 is a transcription factor with a key tumor suppression function in response to cellular stress and DNA damage. *TP53* mutations are frequent in BP, where biallelic loss of *TP53* has been reported in up to 35% of cases, with a dismal outcome [80]. Recently, very low burden of *TP53* mutations has been reported in patients with chronic phase MPN, even many years before diagnosis, with an association with older age [81]. The significance of these very low mutation burdens in relation to the risk of evolution into BP, remains however largely unknown. *PPM1D* mutations are more frequent in cases of therapy related AML and MDS, particularly after cytotoxic agents [82], with an intrinsic chemoresistance to standard therapies [83]. Mutations in *PPM1D*, a regulatory inhibitor of TP53, were recently described in 1.9% of patients with MPN, both within the driver clone and as an independent clone [44]. In Table 1, there is a summary of somatic additional mutations in MPN with their frequency and clinical impact.

## 3. Prognostic Scores in MPN Based on Clinical and Molecular Variables

### 3.1. Polycythemia Vera and Essential Thrombocythemia

For many years, thrombosis prediction in PV and ET relies only on two clinical variables: older age (>60 years) and prior history of thrombosis. More recently, several studies highlighted the possibility that genetic factors contribute to the assessment of thrombotic risk. Unlike patients with PV who almost exclusively carry mutations in *JAK2*, in ET the effect of driver mutations has been extensively evaluated comparing *JAK2*V617F mutated and unmutated patients. Overall, these studies showed that the presence of *JAK2*V617F is associated with a higher risk of thrombosis [88,89,90]. In 2012, on the basis of these pieces of evidence, the International Prognostic Scoring of Thrombosis in ET (IPSET-thrombosis) was developed by adding the *JAK2* mutated status and cardiovascular risk factors (2 and 1 points, respectively) to the classic variables older age (>60 years) and prior thrombosis (1 point each) [91]. A re-analysis of the original IPSET-thrombosis data led to the exclusion of cardiovascular risk factors with the development of a revised IPSET [92] (Table 2) that was subsequently validated in a large independent cohort of ET patients [93]. On the basis of the score, it is suggested that young ET patients without a prior history of thrombosis and lacking the *JAK2*V617F mutation may not need aspirin because of their very low risk of thrombosis. Moreover, it was reported that in this category of ET patients, aspirin could eventually increase the risk of bleeding without reducing the risk for thrombosis [92,94].

Recently, the prognostic relevance of additional somatic mutations was investigated in two large cohorts of PV and ET patients [48]. Upon age-adjusted multivariate analysis of their impact on overall, leukemia-free, and myelofibrosis-free survival, molecular variants associated with adverse outcomes included *ASXL1*, *SRSF2*, and *IDH2* in PV, and *SH2B3*/*LNK*, *SF3B1*, *U2AF1*, *TP53*, *IDH2*, and *EZH2* in ET. Specifically, the presence of at least one of these mutations was associated with inferior overall survival in both PV (median survival, 7.7 vs. 16.9 years) and ET (median survival, 9 vs. 22 years). On the basis of the results obtained from the analysis of 906 molecularly annotated patients from Mayo Clinic and University of Florence cohorts, the authors incorporated genetic data into two new prognostic models: the Mutation-Enhanced International Prognostic Scoring System for PV (MIPSS-PV) and ET (MIPSS-ET) (Table 3) [95]. MIPSS-PV included age >67 years, leukocyte count ≥15 × 10^9^/L, thrombosis history at diagnosis, and the presence of *SRSF2* mutation. Conversely, MIPSS-ET included male sex; age >60 years; leukocyte count ≥11 × 10^9^/L; and the presence of mutations in *SRSF2*, *SF3B1*, *U2AF1*, or *TP53.* A recent sub-analysis of the ET cohort included in the MIPSS-ET demonstrated that *ASXL1*/*RUNX1*/*EZH2* mutations were associated with a decreased risk of arterial thrombosis, suggesting a different underlying biology [96]. These interesting and promising results require further studies to determine their practical role in clinical management.

### 3.2. Primary and Post-PV/ET Myelofibrosis

Currently, two prognostic models that include exclusively hematologic and clinical variables are employed to stratify PMF patients into risk categories with significant differences in overall survival: the International Prognostic Scoring System (IPSS) [97], applicable at the time of diagnosis, and the Dynamic IPSS (DIPSS) [98]. These scores can be applied at any time during the clinical course of PMF, using five clinical variables that independently predict inferior survival: age > 65 years, hemoglobin < 10 g/dL, leukocyte count > 25 × 10^9^/L, circulating blasts ≥ 1%, and constitutional symptoms. Subsequently, the DIPSS was revised to the DIPSS plus [99], including red blood cell transfusion need, platelet count < 100 × 10^9^/L, and unfavorable karyotypes (complex karyotype or sole or 2 abnormalities that included +8, −7/7q-, I (17q), inv (3), −5/5q-, 12p-, or 11q23 rearrangements) [100]. As outlined previously, in addition to driver mutations, more than 80% of patients with PMF harbor genomic variants in myeloid genes, often in multiple combinations [49]. Moreover, in PMF patients, driver and additional mutations have been shown to influence overall and leukemia-free survival, independent of IPSS and DIPSS/-plus [35,56,57]. The prognostic contribution of driver mutations supports the distinction between presence or absence of type 1 *CALR* mutations [101,102], whereas additional abnormalities in *ASXL1*, *SRSF2*, *EZH2*, and *IDH1*/*IDH2* were defined as a high molecular risk (HMR) variable, with a prognostic relevance amplified by the number of those mutations in individual patients [49,54,56]. At the light of these observations, the Molecular Enhanced International Prognostic Score Systems (three-tiered MIPSS70 and four-tiered MIPSS70-plus) were developed using a cohort of patients aged ≤70 years, potentially eligible for allo-HSCT, recruited from multiple Italian centers and the Mayo Clinic, Rochester, USA [103]. The MIPSS70-plus score additionally included unfavorable karyotype defined by any abnormal karyotype other than normal karyotype or sole abnormalities of 20q2, 13q2, + 9, chromosome 1 translocation/duplication, -Y, or sex chromosome abnormality excluding -Y. Both models predicted LFS as well as OS, but MIPSS70-plus seemed to have the best performance in identifying a very high–risk category of patients age ≤70 years, 23% of whom developed acute leukemia likely as a consequence of additional cytogenetic abnormalities. A further revision termed MIPSS70-plus v2.0 [84] incorporated the *U2AF1*Q157 variant as an additional HMR mutation on the basis of previous findings [68]. Additional refinement of risk categories was provided by defining new sex- and severity-adjusted hemoglobin thresholds for anemia (severe for hemoglobin levels of <8 g/dL and <9 g/dL, and moderate for hemoglobin levels of 8 to 9.9 g/dL and 9 to 10.9 g/dL, respectively, for women and men) [104], and by integrating a refined three-tiered cytogenetic risk distribution with the introduction of very high risk (VHR) group; the latter included patients with single/multiple abnormalities of −7, I (17q), inv (3)/3q21, 12p-/12p11.2, 11q-/11q23, or other autosomal trisomies not including +8/+9 [105].

In 2018, all these information regarding the prognostic role of genetic and cytogenetic alterations in PMF prompted the development of a Genetically Inspired Prognostic Scoring System (GIPSS). The latter was exclusively based on molecular (absence of type-1 *CALR* mutations and presence of *ASXL1*, *SRSF2*, and *U2AF1*Q157 additional somatic mutations) and cytogenetic variables [106]. GIPSS was not inferior to DIPSS and MIPSS70-plus in discrimination ability and prediction accuracy. Moreover, these data were validated in a large independent cohort of PMF patients [107].

Notably, all the above models were developed exclusively for patients with a diagnosis of primary MF. When applied to secondary MF, among IPSS, DIPSS, and DIPSS-plus, DIPSS was found to be the most accurate risk stratification model. Further, a four-tiered Myelofibrosis Secondary to PV and ET-Prognostic Model (MYSEC-PM) was specifically developed for secondary MF. This clinical-molecular score included the following variables: hemoglobin level, peripheral blasts, platelet count, age, presence of constitutional symptoms, and *CALR* mutational status [108]. The MYSEC-PM was demonstrated to perform better than IPSS.

In order to accurately predict patients’ outcome following allo-HSCT, the Myelofibrosis Transplant Scoring System (MTSS) was recently formulated for patients with primary and post-PV/ET myelofibrosis. The score variables include: age (≥57 years), performance status, platelet and leukocyte count prior to transplantation (<150 × 10^9^/L and >25 × 10^9^/L, respectively), HLA-mismatched unrelated donor, and *CALR*/*MPL* and *ASXL1* mutational status [109]. Finally, a recent large study based on sequencing of 69 genes in more than 2000 patients with MPN identified a prognostic role for *CBL*, *NRAS*, *RUNX1*, *TET2*, *P53*, *GNAS*, *IDH2*, and *U2AF1* in both OS and LFS. This study led to the creation of a personalized predictive individual model for disease progression and death, integrating a high number of demographics, clinical variables, and genomic variables [44]. However, due to its complexity, clinical application is limited at present.

Prognostic calculators for MYSEC-PM (http://www.mysec-pm.eu/, accessed on 12 July 2021), MIPSS70 (http://www.mipss70score.it/, accessed on 12 July 2021) and MPN personalized risk model (https://jg738.shinyapps.io/mpn_app/, accessed on 12 July 2021) are available online. Table 4 provides a comprehensive overview of the clinical and molecular integrated prognostic scoring systems described in myelofibrosis.

## 4. Impact of Mutational Landscape on Therapeutic Decisions

The remarkable knowledge accumulated in recent years concerning mutational landscape of MPN opened the possibility of developing targeted therapies aiming at specifically interact with cell pathways demonstrated to be dysregulated. The identification of the specific dependence of MPN on JAK/STAT pathway dysregulation, irrespective of the underlying driver mutation, led to the development of small-molecule inhibitors of the JAK family of tyrosine kinases (JAKi). To date, several compounds have been developed that differ in structure, mechanism of action, potency, and kinase selectivity, all being type I inhibitors. It is important to underline that JAKi target the ATP-binding pocket of the JAKs without being selective against mutant JAK protein, thus explaining the clinical efficacy also in *JAK2*-unmutated patients. Ruxolitinib was the first JAK1/2 inhibitor that received approval for myelofibrosis treatment on the basis of the results from the COMFORT-I/II studies [111,112]. Ruxolitinib was effective in reducing spleen volume and alleviating constitutional symptoms, with possible effects on OS. Long term follow-up studies suggested modest reduction of *JAK2*V617F allele burden, with rare cases of molecular remission [113,114]. Subsequently, ruxolitinib was investigated in the RESPONSE [115] and RESPONSE-2 [116] studies that enrolled high-risk PV patients resistant or intolerant to hydroxyurea (according to European Leukemia-Net (ELN) criteria [117]) with and without splenomegaly, respectively. A modest progressive decline in *JAK2* mutant allele burden in patients under ruxolitinib was documented also in the RESPONSE trial, although without clear clinical correlations [118].

Unfortunately, most patients with myelofibrosis on ruxolitinib eventually become resistant to therapy with progression of symptoms and splenomegaly, worsening cytopenias, or evolution to BP. In the COMFORT-II study, among patients responsive to JAKi treatment, less than 50% had chance of maintaining response at five years [113]. Intriguingly, in a recent paper, Newberry et al. [60] demonstrated that 35% of patients treated with ruxolitinib had a clonal evolution after ruxolitinib discontinuation, defined by the acquisition of at least one additional mutation. *ASXL1* mutations was the most frequent, followed by *TET2*, *EZH2*, and *TP53*. The main downside of this study was the lack of a control patients’ cohort [119]. Subsequently, another study, which included a control group of 25 MF patients treated with hydroxyurea, confirmed these observations but also demonstrated that clonal progression is independent of the treatment [120].

The impact of the mutational landscape on treatment outcomes with JAKi has been addressed by a few studies with overall inconsistent findings. A retrospective analysis of the COMFORT-II trial reported that spleen and anemia responses were not correlated with either driver or HMR mutations [111]. Conversely, JAK2V617F allele burden ≥50% was associated with higher spleen response rates to ruxolitinib [121]. In another study evaluating 95 ruxolitinib-treated patients, patients with ≥3 mutations had ninefold lower odds of spleen response and shorter time to treatment discontinuation [122]. Similarly, mutations in *ASXL1* and *CBL* as well as an HMR profile correlated with shorter time to treatment failure [123]. Moreover, loss of spleen response was associated with HMR mutations, whereas the absence of *ASXL1* mutations and > 20% reduction in *JAK2*V617F allele burden at any time during treatment correlated with long-term spleen response [120]. Altogether, these data suggest that although no individual mutations can predict the response to JAKi, particular variants and higher mutational burden may influence the duration of response and hence treatment failure, likely as a consequence of a more aggressive disease. More recently, mutations in the RAS pathways genes (*NRAS*, *KRAS*, *CBL*) were identified as independent predictors of reduced symptom and spleen responses to JAKi [76].

Allo-HSCT remains the only potentially curative therapy for MF patients; despite many improvements, outcomes of allo-HSCT are still burdened by substantial morbidity and high transplant-related mortality and only a minority of patients are eligible for such an intensive procedure. Consequently, insights into molecular mechanisms of MPN pathogenesis have spurred drug development, searching for more effective treatments. In recent years, interferon-α (IFNα), especially in the better tolerate pegylated forms (Peg-IFNα), has emerged as a promising approach in MPN, particularly in PV and ET [124,125,126,127,128,129]. Although IFNα is not currently approved for treatment in ET and PV, consensus guidelines recommend interferon as an option for first-line cytoreduction, particularly in younger or pregnant patients [117]. As suggested by the high rate of reduction of *JAK2*V617F allele burden under Peg-IFNα compared to mutated *CALR*, the treatment appears to selectively target the mutant *JAK2* clone [130]. Enhanced sensitivity of *JAK2*V617F-mutated cells to IFNα may be related to high expression and phosphorylation levels of STAT1 [131]. Interestingly, the presence of concomitant non-driver mutations is associated with smaller mean decreases in *JAK2*V617F allele burden with Peg-IFNα treatment [125]. At this regard, IFNα may have decreased ability to eradicate *TET2* positive clones even when *JAK2*V617F-mutant clone is markedly reduced, indirectly suggesting that the presence of additional mutations can predict responses to IFNα treatment [132]. However, also the dose of the drug seems to impact on the response to treatment; at this regard IFNα at a higher dose in a cohort of 31 *CALR*-positive ET patients induced hematologic responses in all patients with a median reduction of *CALR* mutated allele burden from 41% to 26% [133]. Similar to findings in *JAK2* mutated patients, the presence of *TET2*, *ASXL1*, *IDH2*, and *TP53* additional mutations was associated with poorer molecular responses. In a long-term 83-month follow-up of ET and PV patients treated with Peg-IFNα, median duration of hematologic and molecular response was 66 and 53 months, respectively [134]. Additionally, although in most patients *JAK2*V617F allele burden increased after the first 2 years of treatment, three patients had a complete molecular remission even after discontinuation of therapy. Recently, it was demonstrated that a diplotype spanning the coding region of the *IFNL4* gene influences molecular response to IFNα in PV patients [135]. Nonetheless, disease evolution remained comparable to historical data in patients treated with other therapies, with an 8% transformation rate to MF or BP [134].

Imetelstat, a 13-mer lipid-conjugated oligonucleotide that targets the RNA template of human telomerase reverse transcriptase (TERC), was tested both in MF and ET patients. In 2015, Mayo Clinic investigators reported on 33 patients with intermediate-2 and high-risk MF according to DIPPS-plus score treated with imetelstat (2 h of intravenous infusion; starting dose, 9.4 mg/kg every 1 to 3 weeks) and observed a complete (n = 4; 12%) or partial response in seven patients (21%). Remarkably, all four patients with complete remission experienced reversal of bone marrow fibrosis, and a molecular response occurred in three of these four patients. Responses to imetelstat were correlated with the presence of *JAK2*V617F, *SF3B1*, or *U2AF1* mutations and the absence of *ASXL1* mutation [136]. In another pilot study included 18 ET patients treated with imetelstat, a partial molecular response was detected in seven out of eight *JAK2*V617F-mutated patients. Overall, *JAK2*V617F allele burden was reduced by a median of 71% 3 months after the initiation of treatment, along with allele burden reductions of mutated *MPL* and *CALR* (15–66%) [137]. In the latter cohort of ET patients, additional somatic mutations significantly reduced the depth of response and had an impact on duration of response. Among acquired mutations, *ASXL1*, *EZH2*, and *U2AF1* were responsive to imetelstat, unlike *SF3B1* and *TP53* mutations [138]. Recently, in a phase II study of two imetelstat doses, VAF of *JAK2*V617F, *CALR*, or *MPL* driver mutations by at least 25% was observed in 42.1% of patients in the 9.4 mg/kg arm, and 5.6% of patients in the 4.7 mg/kg arm. Additionally, patients who achieved ≥20% VAF reduction demonstrated higher rates of spleen response, symptom response, and BM fibrosis improvement, along with a longer median OS [139].

Mutations in *IDH1*/*IDH2* and *TP53* are overall uncommon in chronic phase MPN but occur more frequently in BP disease. These findings may have a clinical impact according to the favorable results from studies evaluating ivosidenib and enasidenib (anti-IDH1 and IDH2, respectively) in patients with relapsed/refractory *IDH*-mutated AML [140,141], as well as high response rate of *TP53*-mutated AML to 10-day decitabine [142]. In a mouse model of *JAK2* and *IDH2* co-mutated MPN, combined inhibition of JAK2 and IDH2 (with ruxolitinib and enasidenib, respectively) normalized reduced disease burden to a greater extent than JAK inhibition alone by reversing aberrant gene expression and metabolite perturbation in the hematopoietic stem cell compartment [143]. Murine double minute 2 (MDM2), a key downregulator of TP53, is overexpressed in patients’ CD34+ cells, and treatment with idasanutlin, a second-generation inhibitor of TP53-MDM2 interaction, has been shown to target MPN stem and progenitor cells both alone and in combination with IFNα [144]. On the basis of these data, researchers evaluated idasanutlin in a phase I trial showing hematologic, symptomatic, pathologic, and molecular responses in some patients [145]. Regrettably, the subsequent phase II trial of idasanutlin in HU-resistant/intolerant PV was prematurely terminated due to the high rate of gastrointestinal toxicities. Another MDM2-inhibitor, KRT-232, is being evaluated in patients with *TP53* wild-type MF who are relapsed or refractory to JAKi (NCT03662126).

Anti-apoptotic proteins represent another potential target for MF therapy. At this regard, a promising option is represented by venetoclax, an oral, selective, potent inhibitor of the BCL2 antiapoptotic protein, used in combination with low-dose cytarabine or hypomethylating agents. In a recent trial designed for patients newly diagnosed with AML ineligible for standard induction chemotherapy, azacitidine plus venetoclax was superior to azacitidine alone, also in the subset of *IDH1*/*IDH2*- and *TP53*-mutated patients [146]. Venetoclax in combination with azacytidine/decitabine has also been tested in BP-MPN. In a multicenter series of 32 consecutive cases, complete remission was achieved by 44% of patients and was more likely to occur in the absence of pre-leukemic PV/post-PV MF, complex karyotype, and *NRAS*/*KRAS* mutations, with no correlation with neither *TP53* nor *IDH1*/*IDH2* mutational studies. Importantly, 6/14 patients with complete remission subsequently received allo-HSCT [147].

Although these therapies are promising, BP patients carries a dismal prognosis, with a median survival of ≈6 months; the only possibility of long-term survival is offered by allo-HSCT in the minority of patients who are able to achieve complete remission, or return to chronic phase, with therapy before transplant. Further studies are needed, but these target therapies may represent a valid therapeutic approach also as a bridge to transplantation.

Finally, the prognostic effect of somatic mutations in outcomes following allo-HSCT remains poorly defined. Among 133 patients with PMF or secondary MF receiving HSCT, the presence of *CALR* mutations was associated with better 4-year OS, non-relapse mortality (NRM), and a trend toward lower cumulative incidence of relapse [148]. In survival analysis, patients with mutated *CALR* had the best better prognosis following allo-HSCT, those with *JAK2* or *MPL* mutations had an intermediate prognosis, and TN patients had the worst prognosis. Interestingly, these finding recapitulate those in non-transplant setting [35]. In another retrospective study of 169 MF patients, *CALR*-mutated patients were found to have lower NRM and improved PFS and OS; *ASXL1* and *IDH2* mutations were associated with lower PFS; whereas no impact was observed for TN, *SRSF2*-, or *EZH2*-mutated patients [59]. The presence of somatic mutations in driver and non-driver genes in most MF patients offers the opportunity to use these markers as indicators of minimal residual disease (MRD) after allo-HSCT. Two large studies reported that the persistence of *JAK2*V617F following allo-HSCT was associated with a higher incidence of relapse and a poor OS [149,150]. More recently, in a series of 136 patients, Wolschke et al. demonstrated that patients with detectable driver mutations after allo-HSCT at either day + 100 or day + 180 had a significantly higher risk of relapse at 5 years compared to those in molecular remission [151]. These studies strongly recommended the monitoring of molecular MRD in MF patients after allo-HSCT, helping overall in patient management.

## 5. Conclusions

The summary presented in this review of the molecular abnormalities that harbor patients with MPN, and how these are progressively raising the bar of knowledge of disease pathophysiology as well as improving the management of patients, stand for an exceptional last decade or so of scientific achievements. MPN driver mutations (*JAK2*, *CALR*, and *MPL*) activating JAK-STAT signaling are crucial for MPN pathogenesis. Moreover, additional somatic mutations are detected in more than 50% of MPN cases, particularly in MF, and are associated with disease progression. While in MF many clinical-molecular integrated prognostic models have been developed and routinely used in clinical practice, in PV and ET, the prognostic significance of concomitant somatic mutations is starting to be explored. Yet, there is still much work to do. The category of patients with triple negative MPN is an unmet diagnostic need: why is prognosis so poor for triple-negative MF? What genetic abnormalities do they hide? Are these MPN, or some other disease, for example, are they more akin to myelodysplastic syndrome? The phenomenon of loss of sensitivity to ruxolitinib is a challenging therapeutic dilemma and is known to associate with clonal complexity detected at the time of loss of response. Will we be able to predict it in advance and select the best candidates for alternative treatments? Moreover, the events that promote progression to acute leukemia are largely unknown in terms of what prevents development of appropriate surveillance and early diagnostic criteria. These few examples will hopefully reinforce the interest and promote efforts of the scientific community to reach a full understanding and satisfactorily treatment of MPN.

## Figures and Tables

**Table 1:** Somatic additional mutations in MPN.

Gene	Location	Function	Mutation Type	Frequency	Clinical Consequences	Reference
**DNA Methylation**						
*TET2*	4q24	Evolutionary conserved dioxygenases that catalyze conversion of 5-methyl-cytosine (5-mc) into 5-hydroxymetylcytosine (5-hmc) and promote DNA demethylation	Heterozygous and homozygous loss-of-function mutations mainly in catalytic domain	10–25% of all MPN, including BP	Associated with diseases phenotype; no clear impact on prognosis and thrombosis	[48,49,50,51]
*DNMT3A*	2p23	The encoded protein catalyze 5-methyl-cytosine methylation; regulatory domains allow interactions with histone methyltransferases and histones to influence gene expression	Nonsense/frameshift and missense mutations are described	5–15% of all MPN, including BP	Associated with disease phenotype; detrimental effect in MF and inferior survival	[48,49,50,52]
*IDH1-IDH2*	2q33.3-15q26.1	Isocitrate dehydrogenase 1 and 2 are key metabolic enzymes that convert isocitrate to alpha-ketoglutarate while reducing NADP to NADPH	Mostly heterozygous point missense mutations at residues R132 in IDH1 and R140 or R172 in IDH2	3–6% of MPN; 20–30% of BP	Disease progression to BP and inferior survival	[53,54]
**Histone Modification**						
*ASXL1*	20q11	ASXL1 protein belongs to protein complexes involved in epigenetic regulation of gene expression	Nonsense/frameshift mutations, mostly in exon 12	5–10% of PV and ET; 18–37% of MF and BP	Disease initiation; risk of fibrotic and leukemic progression	[48,57,58,60]
*EZH2*	7q35-36	EZH2 protein is a histone-lysine N-methyltransferase enzyme that participates in histone methylation and transcriptional repression	Heterozygous and homozygous loss-of-function mutations mostly in SET2 domain are described	0–3% of PV and ET; 0–15% of MF and BP	Disease initiation; risk of fibrotic and leukemic progression	[49,62]
**mRNA Splicing**						
*SF3B1*	2q33.1	Involved in pre-mRNA splicing as a component of the splicing factor 3b protein complex	Heterozygous missense point mutations in exons 14–16, mostly involved hotspot K700E	3–5% of PV and ET; 5–8% of MF and BP	Increased risk of fibrotic progression and related with phenotypic change (anemia)	[48,49]
*SRSF2*	17q25.1	The protein is a member of the serine/arginine (SR)-rich family of pre-mRNA spicing factors; in addition to mRNA splicing, the SR proteins are involved in mRNA export from the nucleus and translation	Heterozygous mutations and small in-frame deletions, mostly around hotspot P95	0–3% of PV and ET; 8–22% of MF and BP	Increased risk of leukemic progression and reduced overall survival in MPN	[54,64,67]
*U2AF1*	21q22.3	U2 auxiliary factor 1 is a non-snRNP (small nuclear ribonucleoprotein) protein, member of SR family, required for the binding of U2 snRNP to the pre-mRNA branch site	Heterozygous missense mutations mostly around hotspot S34 and Q157	1–2% of PV and ET; 5–15% of MF and BP	Disease progression and reduced overall survival in MF; related with phenotypic change (anemia)	[68,84]
*ZRSR2*	Xp22.2	Encodes protein associates with the U2 auxiliary factor heterodimer, which is required for the recognition of a functional 3′ splice site in pre-mRNA splicing, necessary during spliceosome assembly	Frameshift/nonsense and missense mutations	0–2% of PV and ET; 5–10% of MF and BP	No clear impact on prognosis	[49,50]
**Cell Signaling**						
*LNK/SH2B3*	12q24	SH2B adapter protein 3 inhibits signaling through cytokine and tyrosine kinase receptors, including JAK2	Mostly heterozygous missense mutations are described as somatic or germline	2–10% of all MPN	Synergy with JAK2V617F; no defined impact on prognosis or thrombosis; may have a role in the context of familial cases of MPN	[48,49,70,72,73]
*CBL*	11q23.3	An adaptor protein that functions as a negative regulator of many pathways that are triggered by activation of cell surface receptors	Mostly homozygous missense substitutions	0–2% of PV and ET; 0–6% of MF and BP	Reduced overall survival in MF, resistance to JAKi; disease progression to BP	[49,50,74,76]
*NRAS* *-KRAS*	1p13.212p12.1	RAS superfamily proteins share a common ability to bind and hydrolyze guanine nucleotides; these proteins are involved in transduction of extracellular signals	Heterozygous missense mutations, particularly in codons 12, 13, and 61	0–1% of PV and ET; 3–15% of MF and BP	Reduced overall survival in MF, resistance to JAKi; disease progression to BP	[76,85,86]
*PTPN11*	12q24.13	Member of the protein tyrosine phosphatase (PTP) family; PTPs are signaling molecules that regulate a variety of processes including cell growth, differentiation, mitotic cycle, and oncogenic transformation	Mostly missense mutations in Src-homology 2 (N-SH2) and phosphotyrosine phosphatase (PTP) domains	0–2% of PV, ET, and MF; 2–5% of BP	Reduced survival in BP	[50]
**Transcription Factors**						
*RUNX1*	21q22.12	It is a transcription factor that forms a complex with the cofactor CBFB (core binding factor B); this complex provide stability to the RUNX1 protein, which is involved in the generation of hematopoietic stem cells and their differentiation	Loss of function missense and frameshift mutations	0–1% of PV and ET; 3–10% of MF and BP	Reduced survival in BP	[44,50]
*NFE2*	12q13.13	It is a component of the NF-E2 complex, essential for regulating erythroid and megakaryocytic differentiation and maturation	Mostly heterozygous frameshift and point mutations	1–3% of PV and ET; 3–10% of MF and BP	No clear impact on prognosis	[78,79]
*PPM1D*	17q23.3	Encodes a member of the PP2C family of Ser/Thr protein phosphatase that regulates the DNA damage response pathway by inhibiting p53 and other tumor-suppressors	Nonsense or frameshift mutations in exon 6	0–2% of all MPN	No clear impact on prognosis or thrombosis; resistance to chemotherapy	[83,87]
*TP53*	17p13.1	Tumor suppressor protein induces growth arrest and apoptosis depending on the physiological circumstances and cell type	Mostly missense mutations	1–3% of PV, ET, and MF; 10–30% of BP	Disease progression to BP and reduced overall survival in all MPN	[50,80,81]

PV, polycythemia vera; ET, essential thrombocythemia; MF, myelofibrosis; BP, blast-phase; MPN, myeloproliferative neoplasms; JAKi, JAK inhibitors.

**Table 2:** Revised IPSET-thrombosis model for essential thrombocythemia.

Risk Category	Clinical Variables	Molecular Variables	Suggested Management
Very low	Age ≤ 60 years, no history of thrombosis	*JAK2* wild type	Management of CV, observation, or low dose ASA, unless contraindicated ^a^.
Low	Age ≤ 60 years, no history of thrombosis	*JAK2* positive	Management of CV and low dose unless contraindicated ^a^. Higher dose ASA ^a^ may be used if presence of CV.
Intermediate	Age > 60 years, no history of thrombosis	*JAK2* wild type	Management of CV risk factors and cytoreductive therapy plus low-dose ASA, unless contraindicated ^a^. Higher dose ASA ^a^ without cytoreductive therapy without CV.
High	Age > 60 years or prior thrombosis	*JAK2* positive	Management of CV risk factors and cytoreductive therapy plus low-dose ASA ^a^.

^a^ ASA is contraindicated if acquired von Willebrand’s disease or active major bleedings are present. IPSET: International Prognostic Score for Essential Thrombocythemia; CV: cardiovascular risk factors; ASA: acetylsalicylic acid (aspirin).

**Table 3:** Clinical-molecular prognostic scores in polycythemia vera and essential thrombocythemia.

Prognostic Score [Reference]	Clinical Variables (Points)	Molecular Variables (Points)	Risk Categories (Points)	Survival *
MIPSS-PV, Tefferi et al. [95]	Leukocyte count ≥15 × 10^9^/L (1); thrombosis history (1); age > 67 years (2)	*SRSF2* mutation (3)	Low (0–1) Intermediate (2–3) High (4–7)	24 13.1 3.2
MIPSS-ET, Tefferi et al. [95]	Leukocyte count ≥11 **×** 10^9^/L (1); age > 60 years (4); male sex (1)	*SRSF2*, *SF3B1*, *U2AF1*, and *TP53* mutation (2)	Low (0–1) Intermediate (2–5) High (6–8)	34.3 14.1 7.9

MIPSS, Mutation-Enhanced International Prognostic Scoring System; PV, polycythemia vera; ET, essential thrombocythemia. * Survival in years.

**Table 4:** Clinical and molecular integrated prognostic scores in myelofibrosis.

Prognostic Score [Reference]	Clinical Variables (Points)	Molecular Variables (Points)	Risk categories (Points)	Survival *
MIPSS70, Guglielmelli et al. [103]	Hemoglobin < 10 g/dL (1) Blasts > 2% (1) Constitutional symptoms (1) Leukocytes > 25 × 109/L (2) Platelet count < 100 × 109/L (2) BM fibrosis ≥ 2 (1)	Non *CALR* type-1 (1) HMR ^a^ = 1 (1) HMR ^a^ ≥ 2 (2)	Low (0–1) Intermediate (2–4) High (5–12)	27.7 7.1 2.3
MIPSS70 plus, Guglielmelli et al. [103]	Hemoglobin < 10 g/dL (1) Blasts > 2% (1) Constitutional symptoms (1)	Non *CALR* type-1 (2) HMR ^a^ = 1 (1) HMR ^a^ ≥ 2 (2) Unfavorable karyotype ^b^ (3)	Low (0–2) Intermediate (3) High (4–6) Very high (7–11)	20.0 6.3 3.9 1.7
MIPSS70 plus v2.0,Tefferi et al. [84]	Hemoglobin 8-10 g/dL (1) Hemoglobin < 8 g/dL (2) Blasts > 2% (1) Constitutional symptoms (2)	Non CALR type-1 (2) HMR^a^+*U2AF1* Q157 = 1 (2) HMR^a^+*U2AF1* Q157 ≥ 2 (3) HR karyotype ^c^ (3) VHR karyotype ^d^ (4)	Very low (0) Low (1–2) Intermediate (3–4) High (5–8) Very high (9–14)	Not reached 10.3 7 3.5 1.8
GIPSS, Tefferi et al. [106]	No clinical variables	Non *CALR* type-1 (1) *ASXL1* mutation (1) *SRSF2* mutation (1) *U2AF1* Q157 (1) HR karyotype ^c^ (1) VHR karyotype ^d^ (2)	Low (0) Intermediate-1 (1) Intermediate-2 (2) High (3–6)	26.4 8.0 4.2 2.0
MYSEC-PM, Passamonti et al. [108]	Hemoglobin < 11 g/dL Blasts ≥ 3% Platelets < 150 × 109/L Constitutional symptoms (2) Age at secondary MF (0.15 point/year)	*CALR* unmutated genotype (2)	Low (<11) Intermediate-1 (11–14) Intermediate-2 (14–16) High (≥16)	Not reached 9.3 4.4 2.0
MTSS, Gagelmann et al. [110]	Platelets < 150 × 109/L (1) Leukocytes > 25 × 109/L (1) Karnofsky PS < 90% (1) Age ≥ 57 years (1) HLA-mismatched unrelated donor (2)	Non *CALR***/***MPL* mutation (2) *ASXL1* mutation (1)	Low (0–2) Intermediate (3–4) High (5) Very high (6–9)	83% ** 64% ** 37% ** 22% **

MIPSS, Mutation-Enhanced International Prognostic Scoring System; GIPSS, Genetically Inspired Prognostic Scoring System; MYSEC-PM, Myelofibrosis Secondary to PV and ET-Prognostic Model; MTSS, Myelofibrosis Transplant Scoring System;. ^a^ High molecular risk (HMR) includes *ASXL1*, *SRSF2*, *EZH2*, *IDH1*/*2*. ^b^ Unfavorable karyotype defined any abnormal karyotype other than normal karyotype or sole abnormalities of 20q2, 13q2, +9, chromosome 1 translocation/duplication, -Y, or sex chromosome abnormality other than -Y. ^c^ High-risk (HR) karyotype includes all the abnormalities that are not VHR and favorable (normal karyotype or sole abnormalities of 20q-, 13q-, +9, chromosome 1 translocation/duplication, or sex chromosome abnormality including -Y). ^d^ Very high risk (VHR) includes single or multiple abnormalities of -7, inv (3), I (17q), 12p-, 11q-, and autosomal trisomies other than +8 or +9. In bold molecular variables. * Overall survival in years; ** 5-year overall survival.

## Data Availability

Not applicable.
